# Microgeography and molecular epidemiology of malaria at the Thailand-Myanmar border in the malaria pre-elimination phase

**DOI:** 10.1186/s12936-015-0712-5

**Published:** 2015-05-13

**Authors:** Daniel M. Parker, Stephen A. Matthews, Guiyun Yan, Guofa Zhou, Ming-Chieh Lee, Jeeraphat Sirichaisinthop, Kirakorn Kiattibutr, Qi Fan, Peipei Li, Jetsumon Sattabongkot, Liwang Cui

**Affiliations:** Department of Anthropology, The Pennsylvania State University, 409 Carpenter Building, University Park, PA USA; Population Research Institute, The Pennsylvania State University, 601 Oswald Tower, University Park, PA USA; Department of Sociology, The Pennsylvania State University, 601 Oswald Tower, University Park, PA USA; Program in Public Health, University of California at Irvine, Irvine, CA USA; Bureau of Vector Borne Diseases, Pra Phuttabhat, Thailand; Mahidol Vivax Research Unit, Faculty of Tropical Medicine, Mahidol University, Bangkok, Thailand; Dalian Institute of Biotechnology, Dalian, Liaoning Province China; Department of Entomology, The Pennsylvania State University, 501 ASI Building, University Park, PA USA

**Keywords:** *Plasmodium vivax*, *Plasmodium falciparum*, Asymptomatic, Thailand, Myanmar, Migration, Spatial disease ecology

## Abstract

**Background:**

Endemic malaria in Thailand continues to only exist along international borders. This pattern is frequently attributed to importation of malaria from surrounding nations. A microgeographical approach was used to investigate malaria cases in a study village along the Thailand–Myanmar border.

**Methods:**

Three mass blood surveys were conducted during the study period (July and December 2011, and May 2012) and were matched to a cohort-based demographic surveillance system. Blood slides and filter papers were taken from each participant. Slides were cross-verified by an expert microscopist and filter papers were analysed using nested PCR. Cases were then mapped to households and analysed using spatial statistics. A risk factor analysis was done using mixed effects logistic regression.

**Results:**

In total, 55 *Plasmodium vivax* and 20 *Plasmodium falciparum* cases (out of 547 participants) were detected through PCR, compared to six and two (respectively) cases detected by field microscopy. The single largest risk factor for infection was citizenship. Many study participants were ethnic Karen people with no citizenship in either Thailand or Myanmar. This subpopulation had over eight times the odds of malaria infection when compared to Thai citizens. Cases also appeared to cluster near a major drainage system and year–round water source within the study village.

**Conclusion:**

This research indicates that many cases of malaria remain undiagnosed in the region. The spatial and demographic clustering of cases in a sub-group of the population indicates either transmission within the Thai village or shared exposure to malaria vectors outside of the village. While it is possible that malaria is imported to Thailand from Myanmar, the existence of undetected infections, coupled with an ecological setting that is conducive to malaria transmission, means that indigenous transmission could also occur on the Thai side of the border. Improved, timely, and active case detection is warranted.

## Background

Although the global situation appears to be improving, malaria remains one of the biggest threats to populations living within the tropical world. The World Health Organization (WHO) estimates that there were 207 million cases of infection and 627,000 malaria-related deaths in 2012 [1]. In much of the Greater Mekong Sub-region (GMS), which includes Cambodia, Laos, Myanmar, Thailand, Vietnam, and China’s Yunnan Province, malaria is now confined to patches in very specific areas. However, the malaria landscape is quite heterogeneous, with many of these patches occurring in hilly, forested areas and along international borders. In Thailand, there has been little to no indigenous malaria in most of the central plains for several decades. In sharp contrast, international borders with Cambodia and Myanmar continue to have endemic malaria. Myanmar has the heaviest malaria burden in the region [2, 3]. The malaria problem along the Thai-Myanmar border is, therefore, often considered a spill-over effect, with population movement from Myanmar being implicated as a driving force in the continued persistence of malaria in the region [2, 4–8].

The ecological, political and demographic situations along this border are conducive to persistent malaria. There are at least three major, almost omnipresent mosquito vector complexes within the border region [9]. The area is heavily forested, mountainous and home to several different ethnic minority groups who are collectively referred to as ‘Hill Tribes’, with the largest group being the Karen. These groups differ from both the Thai and the Burmese in many ways. They have different cultures, languages, demography, ecologies, and subsistence patterns and they all tend to be economically poor. Some live in very remote areas, far from clinics and hospitals, and are therefore sometimes isolated from healthcare services, especially during the rainy season (from May to November). Until very recently, several of these groups have also been at war with the Myanmar government, meaning that they have been isolated along the Myanmar border and many have lived as refugees or displaced persons on the Thai side of the border. Finally, the border is a meeting place of different health policies, with procedures, funding and capabilities that frequently do not overlap. The situation is one in which environmental, socio-political and economic factors combine to form a perfect storm that is favourable to continued malaria transmission.

On the Thai side of the Thailand-Myanmar border, Tak Province has consistently had the highest numbers of reported malaria cases, both *Plasmodium vivax* and *Plasmodium falciparum*, for many years [10]. In recent years, the ratio of *P. falciparum* to *P. vivax* appears to have decreased, with vivax cases now making up the largest proportion of the cases on the Thai side of the border [11]. Within Tak Province, Tha Song Yang district typically has the heaviest malaria burden. However, most malaria surveillance in the region has been through passive case detection (PCD) where cases of malaria are recorded at hospitals, clinics and malaria posts.

As many nations in the GMS are progressing toward regional malaria elimination, a better understanding of malaria epidemiology is essential. The overarching goal of this study was to better understand the epidemiology of malaria on the Thai side of the international border through the use of cross-sectional mass blood surveys coupled with a cohort-based demographic surveillance system. More specifically, the research aim was to determine the microgeographical distribution of malaria cases within the study site and the potential for indigenous, rather than imported, malaria to be present on the Thai side of the border, especially with regard to sub-microscopic malaria and asymptomatic carriers.

## Methods

### Study site and population

**Fig. 1:**
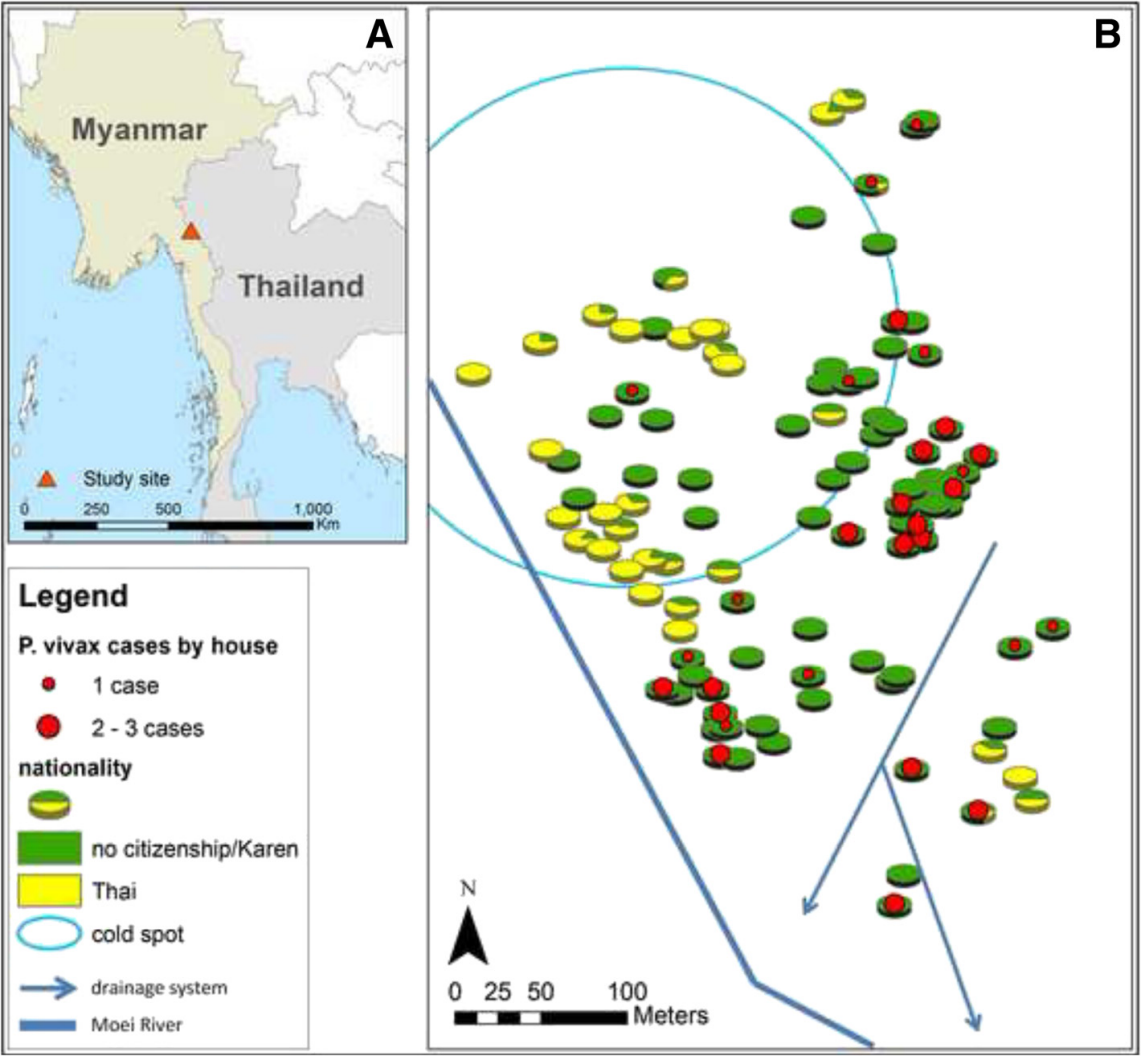
**a** Location of study site; **b** Distribution of *Plasmodium vivax* cases, nationality (a proxy of socio-economic status), and major water systems in the study village. The blue circle indicates a micro-region within the village that consistently has few to no cases (cold spot), detected through the use of scan statistics and SaTScan software

The study site is located along the Moei River on the Thai side of the international border (Fig. 1a). The international border runs along the southwestern quadrant of the study hamlet. It is located on the side of a hill with a difference of over 100 m in elevation from the lowest to the highest household. The full extent of the study site is ~321 m from east to west and 500 m from north to south. A drainage system runs from the northeast to the south-central part of the hamlet (Fig. 1b). A natural spring exists under the hamlet and villagers use it as a source of drinking and bathing water. The water runs through a series of small waterfalls and pools, along the drainage system and ultimately makes its way into the Moei River. Most of the hamlet has been cleared of underbrush and throughout there are eroded waterways and puddles that fill during the rainy season.

### Mass blood and demographic surveys

The protocol of this study has been reviewed and approved by the institutional review boards of the Pennsylvania State University and the Thai Ministry of Health. Informed consent was obtained from the head of each participating household. A full-scale census was conducted and each villager who agreed to participate was given a unique identification code. Demographic variables included sex, self-reported age, occupation, education level, and nationality. Households were also given identification codes and their spatial coordinates were recorded. Household demographic surveys were conducted almost weekly, beginning in October 2011. These surveys noted both additions to a household (through in-migrations or birth) and subtractions from a household (through out-migration or death). Individuals who moved into a household and stayed for a month or longer were coded as in-migrants (from the week that they originally entered the household). When a household member moved out of a household, the study team asked other remaining household members when that person was expected to return. If they were expected to be away for a month or more they are counted as an out-migrant.

Three mass blood surveys (MBS 1–3) were conducted in the study hamlet in July and December 2011, and May 2012. During these surveys, thick and thin blood films were made for each available participant, and these films were immediately checked at the nearest malaria clinic for malaria infection by microscopy. In addition, about 100 μl of finger-prick blood was spotted on Whatman 3-mm filter paper, air dried and stored individually in silicon desiccant for subsequent PCR detection of malaria parasites. Persons with malaria-positive slides were then visited by public health workers and administered anti-malarials according to official Thai protocol. Slides and filter papers were marked with villager and household identification codes and were stored for subsequent analyses (including slide positivity confirmation by expert microscopist, parasitaemia counts, and PCR confirmation of infection).

### Malaria diagnosis

Malaria infections were diagnosed by microscopy using Giemsa-stained thick and thin blood smears at a malaria clinic near the field site. Infections found at this stage were considered to be symptomatic. The results were read again by an expert microscopist at Mahidol University with over 20 years’ experience. For molecular analysis, samples were sent to a collaborating laboratory and a nested PCR method was used to detect malaria infections [12]. Briefly, a 2 × 2 mm disc was punched from each filter paper blood spot and was placed in a centrifuge tube together with 0.5 % saponin in phosphate-buffered saline (PBS, pH 7.0). The primary PCR was performed using genus-specific primers for the rDNA gene, while nested PCR used primers specific for each of the four human malaria species and *Plasmodium knowlesi*, which is known to occur in this region [13]. Infections that were only detected by the expert microscopist or through PCR were considered asymptomatic, based on the assumption that they would be low parasitaemia infections (compared to those that were detected by the field microscopist) and that parasitaemia levels are related to symptoms.

### Risk factor analysis

A mixed effects logistic regression was used to look for statistically significant risk factors for carrying PCR-detected *P. vivax* infections. Because of the small number of *P. falciparum* cases, it was not possible to conduct the same analysis for falciparum malaria. Both household- and individual-level factors were investigated with regard to the individual risk of having a *P. vivax* infection. Individual-level covariates included age group (up to four, five to 14, 15 plus years), sex, nationality (in this case, Thai *versus* those who reported being stateless) and migration status (whether the individual had moved into the study village after the project began). Household-level covariates included: the mean household size for the household with which an individual was associated during the surveys prior to infection; household dependency ratio (ratio of young (age up to 12 years) and elderly (over age 69 years) household members to working age (age 13 to 69) household members); and, whether or not a person had moved in or out of the household during the study period. A random intercept was included for each household to attempt to control for unexplained heterogeneity at the household level.

Several of the covariates in this model act as proxies for social and economic status. People with no nationality are unable to own land, travel freely in Thailand and work most jobs. It is therefore reasonable to associate a lack of nationality with low socio-economic status. The household dependency ratio is also a measure of economic status at the household level. Households with high dependency ratios may be less likely to be able to accrue and sustain surplus finances or food.

Different model specifications were tested, including models that incorporated occupation and education measurements (hindered by high non-response), as well as interaction terms between age and migration, dependency ratio and household size, and nationality and migration. The final model was chosen based on Akaike Information Criterion scores, a measurement of model fit that is penalized by the inclusion of more parameters. Where possible, odds ratios for falciparum risk factors were calculated using contingency tables.

### Spatial analysis

**Fig. 2:**
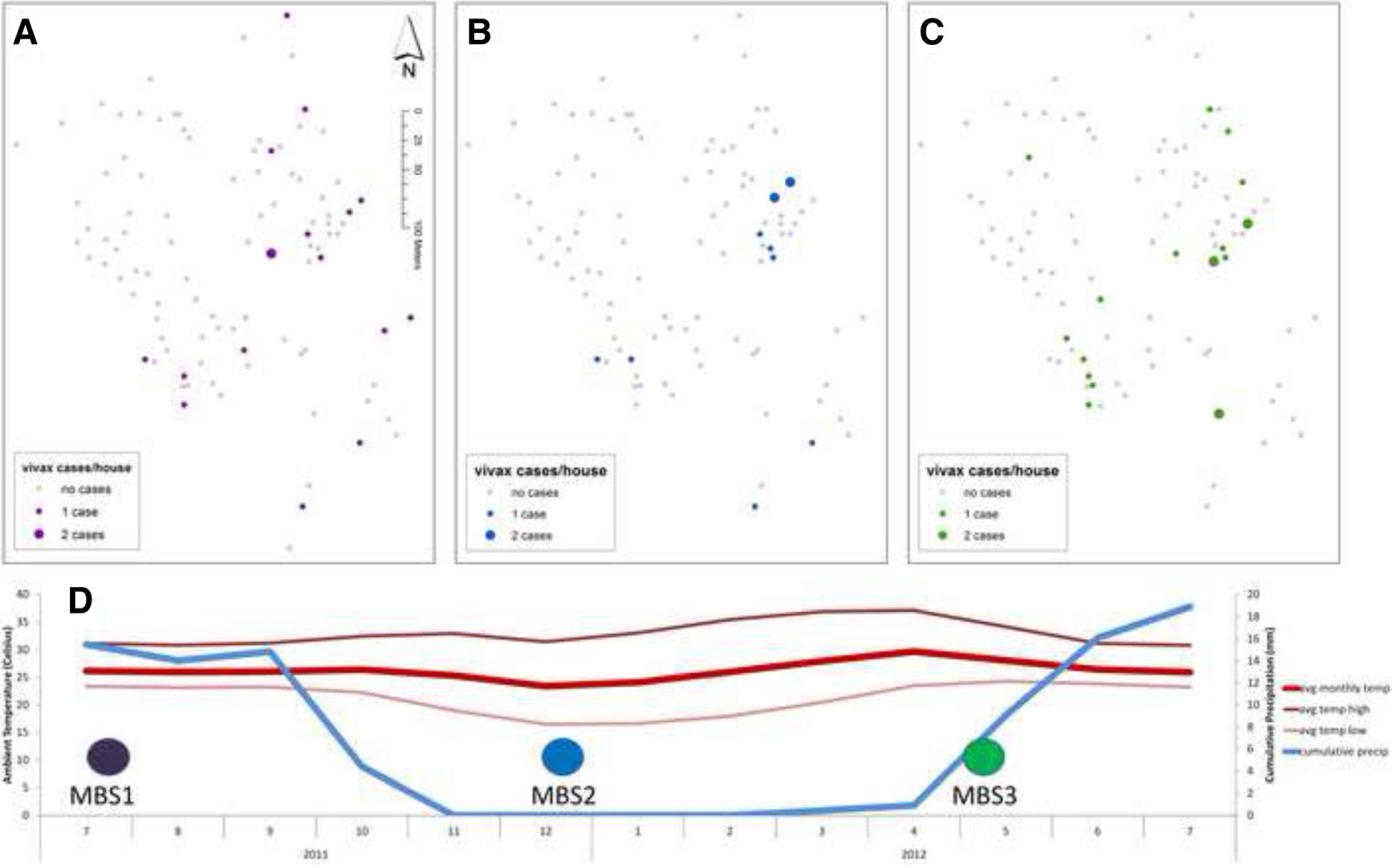
Mass blood survey plots (**a**) MBS 1, **b**) MBS 2, **c**) MBS 3). Households with no cases are indicated by grey squares while households with cases are indicated by graduated circles and colours that correspond to the MBS number. The dates of the MBS are indicated on the timeline beneath (**d**). Meteorological data (**d**) from a nearby weather station are plotted to illustrate seasonality (blue indicates precipitation and red indicates ambient temperature). Village location indicated in Fig. 1a

The distribution of PCR-confirmed *P. vivax* cases were mapped by household for each MBS individually (Fig. 2a-c) and combined (Fig. 1b). CrimeStat III was used to analyse the distribution of cases within each of the MBS [14]. Case dispersion was quantified using the standard distance deviation, a statistic that measures the standard deviation of each point (in this case a household with a vivax case in it) from the mean centre of all households with cases. The data were then tested for spatial clustering (autocorrelation) using three different approaches: Moran’s *I*, join-count statistics, and scan statistics. A k-nearest neighbour approach (using both k = 2 and k = 3) was used to assign spatial weights matrices. For the Moran’s *I* test, cases were standardized in households by household size. For the join-count based test of global autocorrelation the data were transformed into a binary format (1 = a household had 1 or more infections during a given mass blood survey; 0 = a household had no infections during the mass blood survey). Next, SaTScan was used to test for clustering of cases (‘hot spots’) or non-cases (‘cold spots’) [15]. SaTScan uses a moving kernel (circle) of varying sizes that centres on each point (household) in the data set. A discrete Poisson model was used to account for the number of people in a household at each of the MBS. SatScan seeks out kernels with higher numbers of cases or non-cases within the given kernel than would be expected by chance, accounting for the amount of cases that occur outside of the kernel and using a likelihood function to test for statistical significance [16].

## Results

### Demography

At the beginning of the study period, the study site had approximately 512 inhabitants, 48 % of which were male. The study population is an open cohort, with relatively frequent movement in and out of the population over time. The census was updated regularly through household visits and the average (mean) population size during the study period was approximately 494, with a slightly female biased sex ratio (males/females = 0.90). The study population was mostly composed of ethnic Karen, with approximately 78 % of the inhabitants self-identified as Karen, 7 % claimed to be Thai and 16 % did not respond to any of the demographic questions. Conversely, 18 % had Thai citizenship and 66 % had no citizenship (neither Burmese nor Thai). Sixty-six per cent of respondents were illiterate, 13 % had completed primary school and 5 % had completed middle school. Thirty-nine per cent were children and students, 4 % worked in plantations, and 41 % were either not regularly employed or worked in various temporary labour positions.

The population screened during MBS largely followed the age and sex patterns of the census, with an exception of slightly fewer adult males. This pattern is also evident when looking at the cohort that completed all three MBS, with a sex ratio (male/female) in subjects above age 14 of 0.52. Twelve per cent of the participants who were present in censuses were never surveyed during the MBS, most of which (67 %) moved out of the village during the study period. Participation during the three MBS was 74, 79, and 81 %, respectively. The mean and median census ages were 24 and 18, respectively and the mean and median age distributions for MBS closely matched this (25:18, 24:17, 25:18, for mean: median by MBS1, MBS2 and MBS3, respectively). During the study period there were 85 out-migrations and 31 in-migrations (Table 1), with ten of the study participants moving both in and out during the study period. Both in- and out-migration were most pronounced after the agricultural harvest season (between November and March), with out-migration peaking in January.

**Table 1:** Age and sex distribution of study population, PCR-detected infections and participants identified as migrants through the demographic surveillance system

Age Group	Study Population		*P. vivax*		*P. falciparum*		out-migrated		in-migrated	
	male	female	male	female	male	female	male	female	male	female
0 to 4	49	37	2	3	1	0	8	3	4	1
5 to 14	63	76	6	12	3	4	6	9	1	3
15 Plus	150	172	13	14	3	6	32	27	9	13
total	547		50		17		85		31	

### Infections associated with demographic characteristics

Most PCR-detected *P. vivax* infections were in adults (>14 years), with slightly more infections occurring in females than males (Table 1). However, after controlling for the age structure of the population, PCR detected prevalence of both *P. vivax* and *P. falciparum* appears highest in the five to 14 years old age group (approximately 13 vivax and five falciparum infections, per 100 persons, in the five to 14 age group (Table 1)). Interestingly, 94 % of all malaria infections (47/50 *P. vivax* and 16/17 *P. falciparum*) occurred in participants who claimed no nationality while six percent of malaria infections occurred in participants who never responded to questions about demographic characteristics. Malaria infections also appeared to be associated with the levels of education: 70 and 76 % of all *P. vivax* and *P. falciparum* cases, respectively, occurred in illiterate persons and, likewise, 24 and 18 % were in those with only primary school education. Also, 48 and 53 % were in persons with only temporary labour positions, and two and six percent occurred in those who worked in plantations. This latter category consists of highly mobile people, and it is possible that this population was under-sampled because of their mobile nature. Altogether, malaria disproportionally affected people of different demographic groups, with people who were stateless, ethnic Karen, illiterate, and working in only temporary occupations being most heavily affected.

### Malaria diagnosis

This study revealed stark differences by diagnosis approaches. The field microscopist only diagnosed six cases of *P. vivax* and two cases of *P. falciparum*. The expert microscopist diagnosed 18 (18/547 = three percent) *P. vivax* and 16 (16/547 = three percent) *P. falciparum* infections. Six of the vivax cases (6/18 = 33 %) and three of the falciparum cases (3/16 = 19 %) were carrying gametocytes. Parasite counts were lower in cases that were detected by expert microscopy but missed by the field microscopist. The mean asexual *P. vivax* parasite count per 500 white blood cells for *P. vivax* that was undetected by field microscopist was 17.5, compared to a mean *P. vivax* parasite count of 321 in cases detected by both field and expert microscopists. Finally, 55 *P. vivax* and 20 *P. falciparum* cases were detected by PCR. Five cases were mixed infections that were completely missed by microscopy and one *Plasmodium malariae* cases was detected via PCR in a 15 years old male. Five individuals had vivax infections across multiple MBS.

### Risk factors

Using mixed effects logistic regression, potential household- and individual-level factors (modelled as dummy variables) were analysed for increased risks of carrying PCR detectable, blood stage *P. vivax* parasites (Table 2). The most important covariate in the logistic regression model was ‘nationality’. Individuals who had no nationality had over 8½ times the odds of vivax infection (model-adjusted odds ratio, Table 2) and also around 8½ times the odds (unadjusted odds ratio) of falciparum infection.

**Table 2:** Mixed effects logistic model: risk factors for PCR-diagnosed *Plasmodium vivax* infections

Covariates		Coefficient	SE	z value	OR	p value
Individual level	Household level					
Child (0 to 4)						
Child (5 to 14)		0.81	0.59	1.37	2.24	0.1696
Adult (15 plus)		0.29	0.58	0.49	1.33	0.6211
Female						
Male		−0.26	0.34	−0.76	0.77	0.4466
Thai citizenship						
No citizenship		2.16	0.70	3.10	8.68	0.0019
Non-migrant						
In-migrant		0.59	0.76	0.78	1.81	0.4360
	House elevation	−0.14	0.07	−2.07	0.87	0.0381
	Not migrant house					
	Migrant house	−0.16	0.39	−0.42	0.85	0.6782
	Dependency ratio	−0.40	0.66	−0.61	0.67	0.5420

household random intercept (mean ± SE): 9.16 ± 6.17; variance = 0.9327 and standard deviation = 0.9658

There was no statistically significant difference in infections by sex or migration status. Furthermore, household size, household dependency ratio, and living in a household with other migrants did not appear to increase an individual’s risk of infection. There was an effect of household elevation on the risk of individual infection. For each increase in household elevation of 1 m, an individual appeared to have about a 13 % decrease in the odds of vivax infection. Finally, children aged five to 14 years appeared to have the greatest risk of infection (by age), but this effect did not reach statistical significance in the model. Since there are relatively few cases and there is no differentiation between new infections and recurrence, it is important to not over-interpret these results with regard to age groups. Patients with detected vivax parasites across multiple MBS were counted as a single infection in the statistical model.

### Spatial patterns

Maps of PCR-detected *P. vivax* infections by household indicated variation in the spatial distribution of *P. vivax* cases across the MBS (Fig. 2). In particular, cases during the MBS2 (dry season) were fewer and tightly clustered near a few year-round water sources (documented during a follow-up field visit) along the drainage system. Statistically significant spatial clustering of vivax cases was detected by both Moran’s *I* and join-count statistics in MBS2 and MBS3. These tests indicate tight spatial clustering during the dry season (MBS2) and an expansion of cases in the wet season (MBS1 and MBS3). MBS1 was the most dispersed and occurred toward the middle-end of the rainy season (standard distance deviation (SDD): 125.79 m), MBS2 occurred during the dry season (SDD: 116.70 m), and MBS3 occurred as the rainy season was beginning (SDD: 118.37 m). The clusters that are present in the dry season remain in the wet season, but spatial clustering is less evident in the wet season because cases are more widespread throughout the village.

The scan statistics (using SaTScan) gave more detailed, local tests of spatial clustering. The strongest pattern that emerged was the absence of cases in the northwestern quadrant. This area was identified as a cold spot, with a lower than expected number of cases when compared to the rest of the village, in each MBS (MBS1 relative risk (RR) = 0.00; P-value = 0.0310; MBS2: RR = 0.00; P-value = 0.0390; MBS3: RR = 0.17; P-value = 0.0678) and in the combined data (MBS combined: RR = 0.06, P-value <0.0000; indicated in Fig. 1b). A bivariate map showing both the nationality of household members and vivax cases by house revealed an apparent relationship between nationality and infections, with spatial segregation within the village by nationality (Fig. 1b). Cases occurred much more frequently in lower elevation areas, near a major drainage system and year round water sources.

## Discussion

Timely identification and prompt treatment of malaria infected people are both essential to eliminate the transmission source, perhaps especially for regions entering the malaria elimination phase. In many malaria hypo-endemic areas, the presence of asymptomatic patients who can serve as parasite reservoirs requires the use of active (*versus* passive) case detection and sensitive approaches to case detection. Currently, malaria diagnosis by microscopy in Thailand is the norm, with any patient presenting at a malaria clinic or hospital first being diagnosed by microscopy. However, parasite densities in asymptomatic parasite carriers are often below the detection limit of microscopes (normally above 50 parasites/μl blood) [17–20]. Several studies have indicated that low density infections are more easily missed in diagnoses [21, 22]. In addition, the accuracy of microscopic diagnosis is dependent on the skill and training of the microscopist [23–25].

In this study individuals with low parasitaemia were more likely to be missed by the field microscopist when compared to the expert microscopist. This also highlights the need for strengthened programmes of training for microscopists in malaria diagnosis. Around 90 % of malaria infections (both vivax and falciparum) were missed through field microscopy. Another recent paper reports similar discrepancies in malaria diagnoses from the same region [26]. It is likely that cases are missed through microscopic detection throughout this region. Most expert microscopists are located in major laboratories, in major cities, while malaria remains endemic in rural areas. PCR detection is likewise limited to major laboratories. This means that the case numbers that are recorded and reported by Thai public health ministries may underestimate the disease burden. It is important to use extreme caution in basing any inferences, let alone policy, on malaria case numbers when those cases are detected through passive case detection and microscopy alone.

Malaria cases in endemic areas often occur in hotspots, with close spatial associations with vector-breeding habitats, and in certain ‘high-risk’ sub-sets of the population, ‘hot-pops’ (those with higher exposure to vector-breeding habitats). Some previous work in the GMS has indicated that malaria clusters along the international borders and is associated with forests and forest edges [3, 27]. It is also found to be associated with adult males [5, 11, 28, 29]. In addition, migration and population movement are important for malaria epidemiology in this region [4–8, 29, 30]. While this analysis does not refute these suggestions, it certainly describes a different scenario and points to heterogeneities in the malaria ecology of this region, not only at a macrogeographical scale but also at a microgeographical scale.

**Fig. 3:**
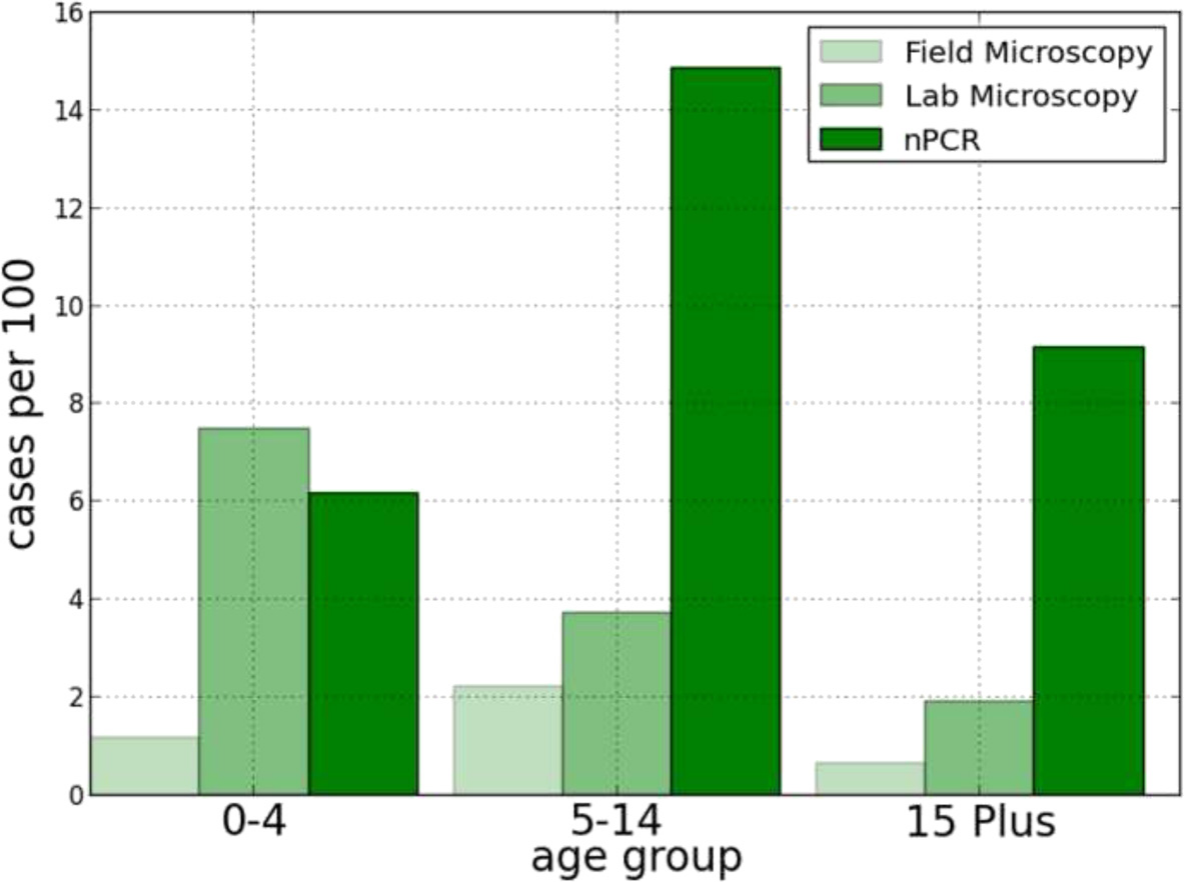
Apparent age distributions of *Plasmodium vivax* cases by method of detection. Individuals with *P. vivax* infections across multiple MBS were only counted once

Whereas none of the age groups showed statistical significance in the logistic model, *P. vivax* infections occurred disproportionately in the five to 14 years age group (Fig. 3). This age pattern has been shown in other studies from the same region and potentially suggests early, relatively widespread exposure to the parasite followed by the acquisition of immunity across the life span. However, several studies in this region have shown that many cases of *P. vivax* infection are in fact recrudescence or relapse which may be triggered by complex factors, including but not limited to host immunological responses or reinfection by other strains or species of malaria [31]. The actual point of infection for vivax is therefore obfuscated by latent, sometimes asymptomatic infections, but the clustering in younger ages suggests that at least some transmission is occurring in or near the hamlet or schools, places where children spend most of their time [11, 29]. This highlights the need to specifically target schoolchildren for malaria elimination in this region.

**Fig. 4:**
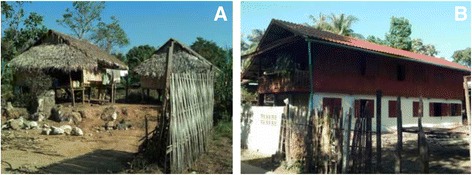
Examples of housing structure from study site. **a**) Typical housing in micro-region where cases are clustered; **b**) Typical housing in area with few to no cases

Furthermore, most studies that have mentioned migration as either a risk factor for infection or as a risk factor for population health have not directly tested for an effect of migration with regard to the risk of malaria infection and have instead been anecdotal. In this investigation it was possible to look directly for associations between migrations and malaria infections, as well as a potential influence of living in a ‘migrant household’, on the risk of malaria infection. This study uncovered no evidence to suggest such an effect, based on the study definition of migration (Table 2). However, the study population could be seen as a transient one, with clustering of cases occurring in displaced persons who have strong cross-border community and family ties, and who have relatively few occupational options on the Thai side of the border. The statistical model indicates a strong correlation between having no citizenship and the risk of vivax infection (Table 2, Fig. 2). Presumably, this risk factor is not directly associated with nationality but with other circumstances and factors ultimately related to the ecology, demography and socio-economic structure of the village. These individuals may also share behavioural traits or norms, including a lack of bed net use or occupational and subsistence strategies, that could differentially put them at greater risk of infection. Aside from their mobile nature, these villagers are relatively poor and live in hastily constructed, open household structures (Fig. 4) which are tightly clustered together at lower elevations and on a landscape that is prone to flooding and holding pools of water.

The spatial ecology of infections in this study further illustrates this pattern, and provides insight into the distribution of cases in a border malaria setting at a micro-level that is the first of its kind to be reported from this specific region. This evidence points towards small pockets of relatively high prevalence of vivax, and potentially falciparum, malaria that may be indigenous to the study hamlet. Cases appear to cluster in specific parts of the study village, which also correlate geographically and socially to ecological (year-round water sources, lower elevation) as well as socio-economic (lack of citizenship and lower socio-economic standing) characteristics that appear related to malaria infections. It is likely that the pattern seen in this short time interval continues over longer stretches of time.

Some of the residents were found malaria-positive by PCR but remained asymptomatic through all MBSs. Five of the participants had vivax infections across consecutive MBS. Therefore some of the villagers are potentially carrying chronic, asymptomatic infections and are therefore potential parasite reservoirs. During the dry season these asymptomatic cases cluster near year-round water sources, while during the wet season they expand. These asymptomatic, potentially long-term infections might be sufficient for sustaining malaria transmission and therefore measures to improve diagnosis and targeted control through active surveillance are essential for malaria elimination.

There are several limitations to this study. Malaria infections were detected in three cross-sectional waves rather than longitudinally and only consist of roughly a year’s worth of time. It is possible that the spatial patterns and the risk factors in this area differ from year to year. The relatively small number of cases and the small size of the population make some analysis difficult. This is a common problem in this region, where low transmission and small population sizes dominate. While this study found no association between migration and risk of vivax infections, the definition and measurement of migration may not be sensitive enough to capture important movement patterns. The study hamlet is located on the international border and residents move back and forth over short time intervals (even within a single day). Such movements are not recorded in this study, can vary by season, and are potentially more common in the study population without citizenship. It is not known whether and how short-term *versus* long-term migration affects malaria introduction and transmission. Finally, in this study it was not possible to differentiate between new infections, recrudescent infections and chronic infections. This limitation means that risk factors should be carefully interpreted as risk factors for being diagnosed as having a vivax infection, given that the point of actual infection is unknown and a participant’s demographic and ecological characteristics (age, migration status, household location) can change over time.

Regardless, these findings have profound implications for malaria work in this region and potentially others. These results suggest that there are a large proportion of missed cases on the Thai side of the border. In this study, many of the cases were detected in children five to 14 years old and in clusters near year-round water sources on the Thai side of the border. Also, most of the infections in this study were submicroscopic and several appeared to be chronic. Malaria vector species are known to inhabit the Thai side of the border and many people carrying parasites in their blood are unlikely to be detected through passive case detection and microscopy. Importation of malaria is therefore possible, but not necessary, for continued transmission within Thailand. Such reservoirs will remain crucial targets in these efforts as national and sub-national programmes begin to move towards elimination.
